# Renal Function following Fluorescein Angiography in Diabetic Patients with Chronic Kidney Disease

**DOI:** 10.18502/jovr.v18i2.13183

**Published:** 2023-04-19

**Authors:** Nazanin Ebrahimiadib, Shaghayegh Hadavand Mirzaei, Hamid Riazi-Esfahani, Manouchehr Amini

**Affiliations:** ^1^Eye Research Center, Farabi Eye Hospital, Department of Ophthalmology, Tehran University of Medical Sciences, Tehran, Iran; ^2^Department of Nephrology, Tehran University of Medical Sciences, Tehran, Iran; ^5^https://orcid.org/0000-0002-4618-0866

**Keywords:** Acute Kidney Injury, Chronic Kidney Disease, Diabetic Retinopathy, Fluorescein Angiography, Nephropathy, Serum Creatinine

## Abstract

**Purpose:**

To evaluate the effect of fluorescein dye usage on renal function in patients with diabetic retinopathy (DR) and chronic kidney disease (CKD).

**Methods:**

Diabetic patients with retinopathy who were candidate for fundus fluorescein angiography (FA) were evaluated for serum creatinine and urea levels within five days prior to performing the FA. Serum creatinine levels of 1.5 mg/dl or more in males and 1.4 mg/dl or more in females were both identified as CKD and were included in the study. An increase of 0.5 mg/dl or 25% in creatinine after FA was considered as contrast-induced acute kidney injury (AKI). Estimated glomerular filtration rate (eGFR) was also calculated for all patients using a CKD-Epi formula. CKD grading was determined based on eGFR values.

**Results:**

Forty-two patients agreed to participate, of which 23 (54.8%) were male. Seventeen patients were identified with grade 3a or lower CKD, 12 with grade 3b, 11 with grade 4, and two with grade 5 CKD. Considering all grades of CKD, the mean blood urea before and after angiography was 58.48 ± 26.7 and 57 ± 27.81 mg/dl, respectively (*P* = 0.475). The mean serum creatinine before and after the test was 1.89 ± 1.04 and 1.87 ± 0.99 mg/dl, respectively (*P* = 0.993). The mean eGFR before and after the test was 44.024 ± 23.5447 and 43.850 ± 21.8581** mL/min/1.73 m^2^** *(P = 0.*875).

**Conclusion:**

According to the findings of this study, FA does not seem to further deteriorate kidney function in patients with diabetic associated CKD.

##  INTRODUCTION

Fundus fluorescein angiography (FA) is a valuable method to evaluate microvascular complications in patients with diabetic retinopathy (DR). This procedure involves injection of fluorescein dye, which is a non-ionizing contrast, intravenously. Fluorescein dye is metabolized by the kidneys and excreted in the urine during the first 48–72 hr after injection.^[1]^ During this time, contrast-induced nephropathy usually manifests.^[2]^

Considering that concomitant diabetic nephropathy is possible in patients with DR, there is a concern as to whether fluorescein usage can deteriorate the kidney function further.^[1,2,3,4]^ Chronic kidney disease (CKD) is defined as a gradual decline in renal function which is initially subclinical. Contrast-induced acute kidney injury (AKI) is defined by an increase in serum creatinine of ≥ 0.5 mg/dl or 25% from the baseline that occurs around 48 hr after contrast administration.^[1]^

Although renal side effects have been attributed to fluorescein usage in previous studies, there is still no consensus in this regard.^[5,6]^Some angiography centers require a nephrology consult, in addition to blood and urine workup, before preceding to FA in patients with CKD. Apart from the burden of cost and time on patients and healthcare system, referral of these patients for additional testing may cause a delay in diagnosis and treatment of the eye condition.

In this study, we are going to determine whether the fluorescein can cause contrast-induced AKI in diabetic patients with CKD who are candidates for fundus FA.

##  METHODS

This prospective study was performed in the retinal imaging section of Farabi Eye Hospital from January 2019 until January 2020. The study protocol was reviewed and approved by the ethics committee of Tehran University of Medical Sciences under approval number 96014334034. Prior to recruitment, all patients were informed of the details of the study through verbal and written communication. According to the Helsinki Declaration, a written consent was also obtained from patients.

Patients with DR who were referred to the imaging section for performing FA to confirm the stage of retinopathy were routinely asked for a history of renal dysfunction. Those with a positive history were sent for blood test to determine urea and creatinine levels. If creatinine levels were ≥ 1.5 mg/dl in men and ≥ 1.4 mg/dl in women, they were included in the study.^[7]^The glomerular filtration rate (GFR) (ml/min/1.73 m^2^) was calculated for all patients based on the Chronic Kidney Disease Epidemiology Collaboration (CKD-EPI) equation.^[8]^The CKD grading was determined based on the eGFR levels. According to the Kidney Disease Outcomes Quality Initiative guidelines, eGFR of ≥ 90 ml/min per 1.73m ${}^{2}$ is considered stage 1 CKD, 60–89 stage 2, 30–59 stage 3 (45–59 stage 3a and 30–44 stage 3b), 15–29 stage 4, and < 15 would be considered stage 5 CKD.^[8]^

The process of FA involved an injection of 2.5 ml of 10% fluorescein sodium solution (1g/5cc) [Sterop, Belgica] into the antecubital vein access in 5 s. For at least 10 min after the dye injection, fundus images were captured by Heidelberg retina angiography with confocal SLO (Heidelberg Engineering, Heidelberg, Germany). Patients were then monitored for about 15 more minutes for any complications. Blood urea and creatinine levels were retested 48–72 hr after the fluorescein injection. Serum creatinine levels, before and after contrast administration, were compared and if increased by ≥ 0.5 mg/dl or 25% from the baseline were considered as AKI.^[2]^

Moreover, patients who had received contrast material for imaging during the prior two months or those with kidney failure due to another cause, final stage of renal dysfunction requiring dialysis, chronic heart failure, pregnancy and breastfeeding, history of sensitivity to the contrast agent, consumption of nonsteroidal anti-inflammatory drug, angiotensin receptor blocker, angiotensin converting enzyme inhibitor, or intravenous diuretic were all excluded from the study.

Data analysis was performed using the SPSS software version 18 (SPSS, Inc., Chicago, IL, USA). Alterations of the variables with normal distribution was analyzed using paired sample *t*-test, and Wilcoxon singed-rank test was used to compare nonparametric variables. *P* < 0.05 was considered as significant.

##  RESULTS

We included 42 patients, with the mean age of 58.1 years. Of them, 23 (54.8%) were male. Regarding the CKD grading, 17 patients were identified with grade 3a or lower CKD, 12 with grade 3b, 11 with grade 4, and two with grade 5.

Overall mean urea in patients before and after angiography was 58.48 ± 26.7 and 57 ± 27.81 mg/dl, respectively (*P* = 0.475).

The mean serum creatinine levels were 1.89 ± 1.04 and 1.87 ± 0.99 mg/dL as the pre- and post-angiography values (*P* = 0.993). The mean eGFR before and after the test was 44.024 *±* 23.5447 and43.850 *±* 21.8581mL/min/1.73 m^2^** (*P* = 0.875) [Table 1].

Differential changes in eGFR before and after FA in all stages of CKD was not significant [Table 2].

##  Discussion

Findings in our study showed that FA does not exacerbate kidney dysfunction (AKI) in diabetic patients with an already reduced GFR (CKD). Therefore, for patients with diabetic nephropathy in this instance who are candidates for FA, nephrology consult or additional tests before and after fluorescein injection are not required.

In a retrospective study by Kameda et al, patients' serum creatinine level within one month before and after FA were investigated to calculate the estimated glomerular filtration rate (eGFR). They categorized patients with eGFR of < 60 ml/min/m^2^ into three grades of severity from grade 3 to 5 CKD. None of these subgroups showed a significant alteration in eGFR following fluorescein injection.^[3]^

In another study by Chung et al, patients were divided into three categories based on serum creatinine levels before angiography; including low ( < 1.5 mg / dl), moderate (1.5–2 mg/dl), and high ( > 2 mg/dl).^[9]^Then, the effect of FA on renal function was evaluated in these patients. However, the actual timing that they tested the serum creatinine levels before and after FA was indeterminate, especially as there were some delayed measurements after FA, which might have influenced the omission of cases with AKI. AKI definitions are based on changes up to a maximum of seven days following the presumed insult.^[10]^They also included patients with other causes of renal dysfunction other than diabetes. They reported a significant change in serum creatinine levels in the high-risk group and suggested caution be applied for these patients.^[9]^

**Table 1:** Values of blood urea and creatinine levels and estimated GFR, before and after fluorescein angiography in patients with chronic kidney disease.


**Grades of CKD**	**eGFR (mL/min/1.73 m^2^)**		***P*** **-value**
	**Pre**	**Post**	
G1	98.50 ± 3.50	89.70 ± 11.05	0.309
G2	67.13 ± 7.98	64.20 ± 7.86	0.430
G3a	50.62 ± 5.12	51.25 ± 10.38	0.822
G3b	37.65 ± 5.44	40.54 ± 8.03	0.133
G4	21.74 ± 4.52	22.18 ± 4.23	0.640
G5	10.95 ± 5.02	10.50 ± 4.10	0.614
	
	
CKD, chronic kidney disease; eGFR, estimated glomerular filtration rate			

**Table 2:** Alteration of eGFR following fluorescein angiography.


	**Mean ± SD (range)**	**Median (range)**	**95% CI**		***P*** **-value**
		**Lower**	**Upper**	
Urea (mg/dL)	Pre	58.48 ± 26.7 (0.7–6)	55 (23 to 134)		
	Post	57 ± 27.81	46 (21 to 126)		
	Change	–1.48 ± 13.27	1.5 (–35 to 25)	–5.61	2.66	0.475
Creatinine (mg/dL)	Pre	1.89 ± 1.04 (0.7–6)	1.65 (0.7 to 6)		
	Post	1.87 ± 0.99 (0.7–5.9)	1.55 (0.7 to 5.9)		
	Change	–0.02 ± 0.25	0 (–1.1 to 0.4)	–0.1	0.06	0.993
eGFR by CKD-Epi (mL/min/1.73 m^2^)	Pre	44.024 ± 23.54 (7.4–102)	55 (7 to 98)		
	Post	43.850 ± 21.86 (7.6–97.1)	46 (7 to 95)		
	Change	–0.174 ± 22.69	0.17 (–40 to 67)	–2.039	2.38	0.875
	
	
SD, standard deviation; CI, confidence interval; CKD-Epi, chronic kidney disease epidemiology collaboration equation; CKD, chronic kidney disease; eGFR, estimated glomerular filtration rate						

In another study by Alemzadeh et al on 44 diabetic patients, serum creatinine levels showed a significant increase 72 hr after FA in 20% of patients. In contrast to studies by Kameda and Chung, Alemzadeh study showed that an increase in serum creatinine and AKI can happen secondary to FA. However, their patients were not categorized in terms of kidney damage.^[11]^ In our study, although the mean creatinine level was higher as compared to the Alemzadeh's study both before and after FA, the dose of injected fluorescein was 250 mg which was half of the dose used in their study.

Kidney damage and increase in serum creatinine following the use of contrasts in susceptible patients has been attributed to vasoconstriction, which reduces blood flow to the medulla. Of note, renal blood flow auto regulation is defective in patients with CKD. Identifying high-risk patients and preventing the occurrence of AKI is critical,^[12]^especially as diabetes mellitus is the leading cause of propensity toward renal dysfunction.

It is noteworthy that most kidney damage related to contrast agents has been due to iodinated contrast agents, while fluorescein is a non-iodinated one.^[13,14,15,16]^ However, in a recently published article with a retrospective design, fluorescein was found to play a role in the progression of nephropathy. Nevertheless, the authors were not sure of the clinical significance of their result due to two reasons. Firstly, under normal circumstances fluctuations of up to 15% are possible each time the serum creatinine level is tested. The second reason is related to the criteria used; in a patient with AKI, creatinine-based formulas are prone to overestimating the eGFR. Therefore, due to uncertainty about creatinine levels, their conclusion about the effect of the fluorescein on nephropathy is dubious.^[17]^

Our study was a prospective study on patients with DR and CKD. We found no significant effect on renal function shortly after performing FA. We used half of the recommended dosage of fluorescein and could still obtain good-quality images of fundus angiography. Nephrology consults and further evaluation for these group of patients seem to be unnecessary.

There are two major limitations for our study. Firstly, the relatively small number of cases. Secondly, we did not categorize our patients according to the multiple types of medications used by each patient; such as oral hypoglycemic agents, diuretics, or statins. However, as overall we did not observe fluorescein injection causing any AKI, lack of such data might not be affecting the results.

##  Financial Support and Sponsorship

None.

##  Conflicts of Interest

None.
